# Quinolinate Phosphoribosyltransferase is an Antiviral Host Factor Against Hepatitis C Virus Infection

**DOI:** 10.1038/s41598-017-06254-4

**Published:** 2017-07-19

**Authors:** Zhilong Wang, Yanhang Gao, Chao Zhang, Haiming Hu, Dongwei Guo, Yi Xu, Qiuping Xu, Weihong Zhang, Sisi Deng, Pingyun Lv, Yan Yang, Yanhua Ding, Qingquan Li, Changjiang Weng, Xinwen Chen, Sitang Gong, Hairong Chen, Junqi Niu, Hong Tang

**Affiliations:** 1 The Joint Laboratory for Translational Precision Medicine of Wuhan Institute of Virology, Chinese Academy of Sciences and Guangzhou Women and Children`s Medical Center, Wuhan Institute of Virology, Chinese Academy of Sciences, Wuhan, Hubei 430071 China; 2 The Joint Laboratory for Translational Precision Medicine of Wuhan Institute of Virology, Chinese Academy of Sciences and Guangzhou Women and Children`s Medical Center, Guangzhou Women and Children’s Medical Center, Guangzhou, 510623 China; 3 CAS Key Laboratory of Infection and Immunity, Institute of Biophysics, Chinese Academy of Sciences, Beijing, 100101 China; 4 Institut Pasteur of Shanghai, Chinese Academy of Sciences, Shanghai, 200031 China; 5 Harbin Veterinary Research Institute, Chinese Academy of Agricultural Sciences, Heilong Jiang, 150001 China; 6 Department of Hepatology, The First Hospital of Jilin University, Changchun, Jilin 130021 China; 7 University of Chinese Academy of Sciences, Beijing, 10049 China

## Abstract

HCV infection can decrease NAD^+^/NADH ratio, which could convert lipid metabolism to favor HCV replication. In hepatocytes, quinolinate phosphoribosyl transferase (QPRT) catabolizes quinolinic acid (QA) to nicotinic acid mononucleotide (NAMN) for *de novo* NAD synthesis. However, whether and how HCV modulates QPRT hence the lipogenesis is unknown. In this work, we found QPRT was reduced significantly in livers of patients or humanized C/O^Tg^ mice with persistent HCV infection. Mechanistic studies indicated that HCV NS3/4A promoted proteasomal degradation of QPRT through Smurf2, an E3 ubiquitin-protein ligase, in Huh7.5.1 cells. Furthermore, QPRT enzymatic activity involved in suppression of HCV replication in cells. Activation of QPRT with clofibrate (CLO) or addition of QPRT catabolite NAD both inhibited HCV replication in cells, probably through NAD^+^-dependent Sirt1 inhibition of cellular lipogenesis. More importantly, administration of CLO, a hypolipidemic drug used in clinics, could significantly reduce the viral load in HCV infected C/O^Tg^ mice. Take together, these results suggested that HCV infection triggered proteasomal degradation of QPRT and consequently reduced *de novo* NAD synthesis and lipogenesis, in favor of HCV replication. Hepatic QPRT thus likely served as a cellular factor that dampened productive HCV replication.

## Introduction

HCV infects at least 185 million people worldwide^1^. HCV infection is often asymptomatic^2, 3^ and most of the infected adults can develop chronic infection^4^. More devastating, a significant portion of chronic hepatitis C (CHC) progresses to more severe hepatic pathology, including steatosis, cirrhosis and hepatocellular carcinoma^5^. Besides the escape mutations, impaired and/or insufficient immune response to HCV is believed to account for HCV persistent infection^6^, but the precise mechanisms are still obscure.

QPRT catalyzes quinolinic acid (QA), the metabolite of L-Trp in kynurenine (kyn) pathway (KP), to nicotinic acid mononucleotide (NAMN) for *de novo* synthesis of nicotinamide adenine dinucleotide (NAD), in both prokaryotes and eukaryotes^7, 8^. A tight control of QA level by QPRT is thus required, since hyper QA is often associated with neuropathology and autoimmune diseases^9^. On the other hand, NAD plays important roles in various cellular processes. NAD serves as both a coenzyme for hydride-transfer enzymes and a substrate for an array of NAD^+^-dependent enzymes, including Sirtuins, the type III protein lysine deacetylases^10^. Sirtuins, notably SIRT1, primarily involves in inhibition of adipogenesis and enhancement of lipolysis^11^. Unlike other known protein deacetylases, which simply hydrolyze acetyl-lysine residues, the sirtuin-mediated deacetylation reaction couples lysine deacetylation to NAD hydrolysis. This hydrolysis yields O-acetyl-ADP-ribose, the deacetylated substrate and nicotinamide, itself an inhibitor of sirtuin activity. The dependence of sirtuins on NAD links their enzymatic activity directly to the energy status of the cell via the cellular NAD/NADH ratio, the absolute levels of NAD, NADH or nicotinamide or a combination of these variables. HCV replication can decrease the NAD^+^/NADH ratio by down-regulation of both the expression and enzymatic activity of SIRT1, which converts lipid metabolism to favor HCV replication in hepatocytes^12, 13^. Moreover, NAD is also suggested to play an anti-viral role through PARP superfamily members^14–18^. However, how HCV affects NAD metabolism has not been fully elucidated.

This work has shown that QPRT is significantly reduced by HCV infection in livers of patients or humanized C/O^Tg^ mice with persistent HCV infection. Mechanistic studies indicate that HCV NS3/4A can drive proteasomal degradation of QPRT facilitated by the E3 ubiquitin ligase Smurf2. Furthermore, overexpressed QPRT can inhibit HCV replication relying on its enzymatic activity. Administration of QPRT agonist or direct NAD treatment can both inhibit HCV replication both *in vitro* and *in vivo*. Together, this work has identified that HCV reduces NAD *de novo* synthesis by promoting QPRT degradation. QPRT thus provides the cytosolic immunity against HCV infection, and QPRT-NAD pathway may serve as a potential target to develop therapeutics against chronic hepatitis C.

## Results

### QPRT underwent proteasomal degradation by HCV infection

**Figure 1:**
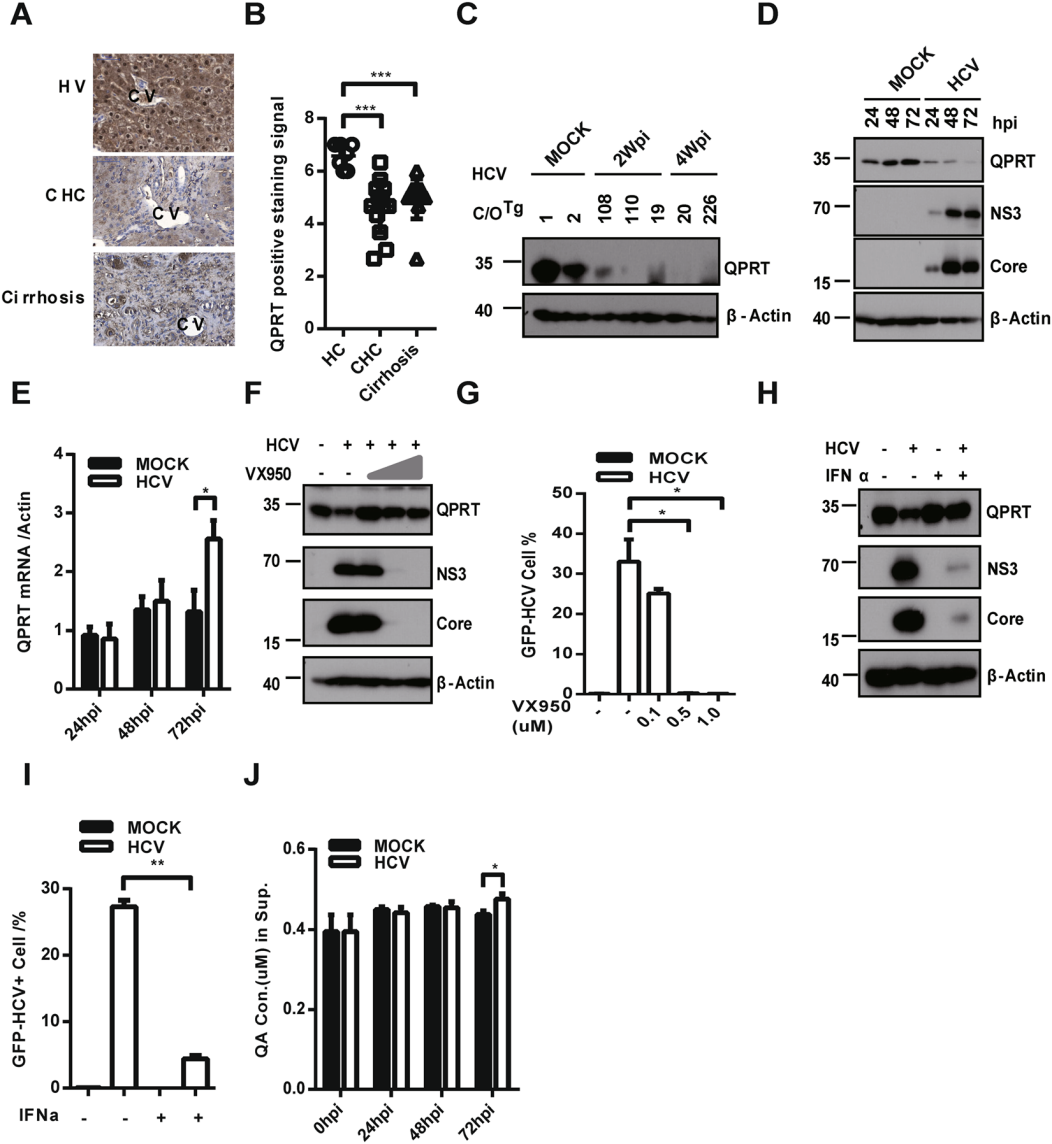
HCV infection reduced QPRT expression and NAD catabolism. (**A**) IHC staining of QPRT (dark brown) was performed and (**B**) the scores of QPRT positive areas were scored on liver biopsies of healthy donor (HV), chronic HCV infection (CHC) and HCV induced cirrhosis (Cirrhosis). cv, central vein, scale bars, 50 μm. (**C**) Expression levels of hepatic QPRT were measured by immunoblotting after C/O^Tg^ mice were mock infected or infected with HCV J339EM for 2 (#19, 108, 110) and 4 weeks (#20, 226). β-actin was used as a loading control. (**D**) Decay of QPRT was assessed by immunoblotting after Huh7.5.1 cells were mock or infected with HCV for indicated time. HCV NS3 and Core proteins were detected by immunoblotting for HCV replication. (**E**) QPRT mRNA was detected by qRT-PCR and plotted as the ratio to actin. After Huh7.5.1 cells were infected with HCV for 6 h, the increasing concentration of VX950 (**F–G**) or 1000 U/mL IFNα-2b (**H–I**) was added for 48 h. Cells were detached and (**F,H**) detected by immunoblotting for QPRT, NS3 and Core proteins, and (**G,I**) FACS measured for GFP positivity. (**J**) Huh7.5.1 cells were infected with HCV J399EM for indicated time, and extracellular QA was analyzed by LC-MS/MS. Full-length gels and blots are included in the Figure S5. Error bars were SEM of three independent experiments, student *t* test, *P < 0.05; **P < 0.01, ***P < 0.001.

To determine whether QPRT plays any role in HCV infection, we first measured the expression of QPRT in liver biopsies of CHC patients (Table S1, 9 of 11 CHC and 6 of 13 cirrhotic patients chosen for serum HCV RNA > 100 IU/mL). Immunohistochemical (IHC) analysis showed in general a much less QPRT expression in CHC and cirrhotic patients (Fig. 1A,B). A closer inspection of disease progression revealed an inverse correlation between HCV genome copies and hepatic QPRT expression for patients at disease grades G2 and G3, respectively (Figure S1A). Too few tissue samples available for G1 grade or massive tissue damage and inflammation at G4 grade prevented a justifiable correlation between HCV replication and QPRT levels. Nevertheless, these results suggested that HCV replication might cause a decrease of QPRT protein levels at the chronic phase (G2 and G3) of hepatitis C.

To better correlate the QPRT levels to HCV infection *in vivo*, we re-assessed the hepatic QPRT levels in the previously characterized C/O^Tg^ mice^19^, either 2 (acute infection) or 4 weeks (persistent infection) after HCVcc inoculation (Figure S1B for hepatic HCV RNA copies). Immunoblotting of HCV infected C/O^Tg^ livers indicated that QPRT expression was greatly reduced compared to the mock infection (Fig. 1C). To delineate how HCV would affect QPRT levels, we further performed *in vitro* analysis. HCV infection of Huh7.5.1 cells caused a decrease of QPRT protein in a time dependent manner (Fig. 1D). In this condition, the level of QPRT mRNA remained constant, and even slightly increased at 72 h after HCV infection (Fig. 1E). Therefore, HCV likely reduced QPRT at the post-translational level. Finally, inhibition of HCV replication in Huh7.1.5 cells by either NS3/4 A protease specific inhibitor VX950 (Fig. 1F) or IFNα (Fig. 1H) attenuated the declining tendency of QPRT. Thus, QPRT protein level would specifically respond to HCV replication. Both treatments effectively reduced viral replication, as measured by the drastic reduction of NS3 and core (Fig. 1F,H), and the reduced percentage of GFP^+^ Huh7.5.1 cells (Fig. 1G,I). In HCVcc J339EM, NS5A was fused with EGFP that was expressed along HCV replication^19^. Of note, the percentage of GFP positive cells was in good linearity to HCV genome copies (Figure S1C), we routinely used the percentage of GFP+ cells to represent the replication efficiency of HCV in Huh7.5.1 cells. Finally, less QPRT would lead to a reduced consumption of its enzymatic substrate QA. Mass spectrometric analysis showed that QA concentration in the supernatant was indeed increased by ~9% at 72 h post HCV infection of Huh7.5.1 cells (Fig. 1J). Therefore, QPRT can be reduced by HCV infection, indicative of its role in regulation of HCV infection. It is thus desirable to delineate the biological significance of QPRT in regulation of HCV replication.

### HCV promoted QPRT proteasomal degradation through Smurf2

**Figure 2:**
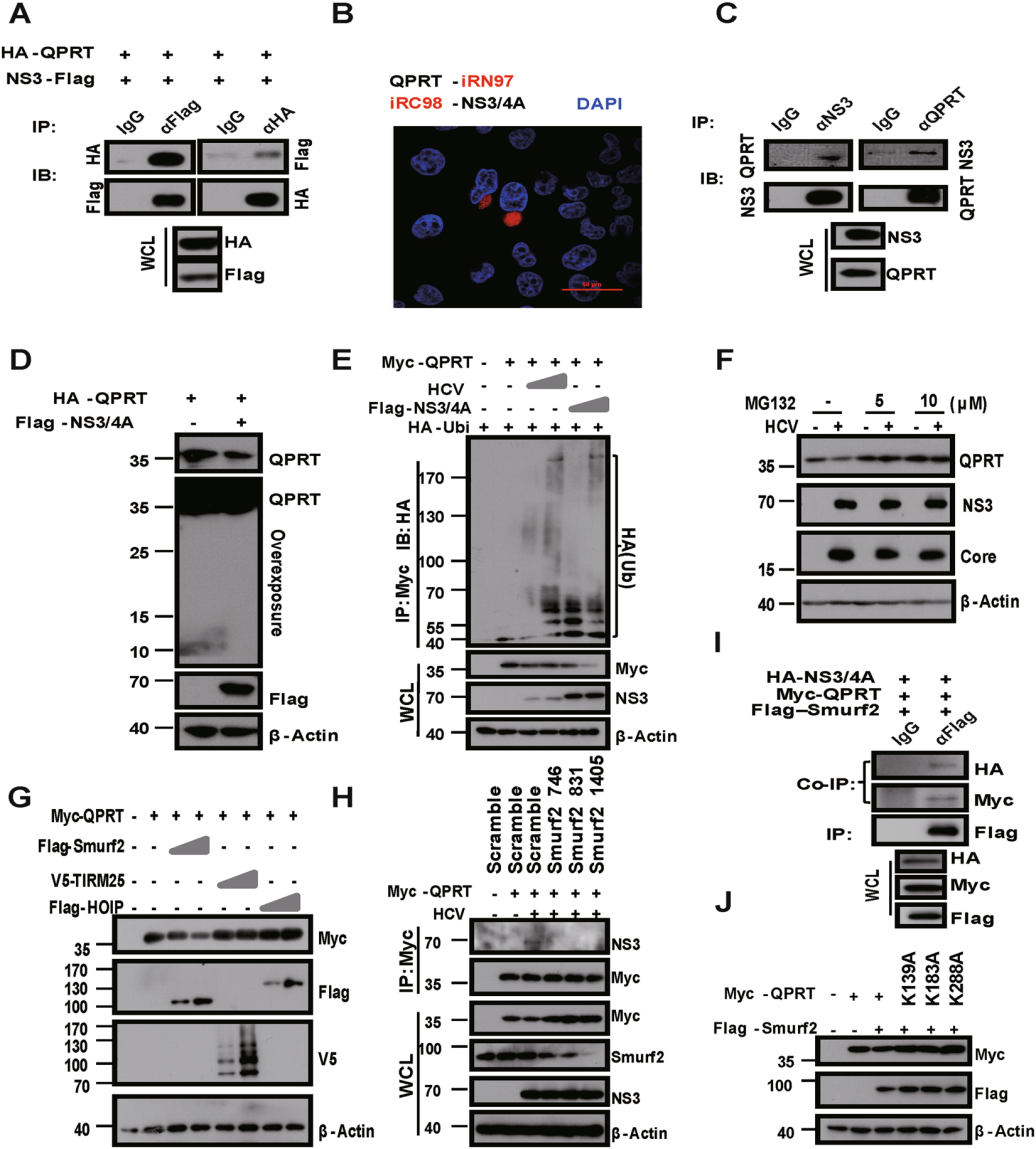
HCV NS3 triggered Smurf2 mediated proteasomal degradation of QPRT. (**A**) Reciprocal co-IP to assess the molecular interaction between the co-expressed NS3-Flag and HA-QPRT in 293T cells using the indicated themes. Mouse IgG was used as a negative control. (**B**) Interaction between co-transfected QPRT-iRN97 and iRC98-NS3/4 A in HeLa cells were assessed for BiFC signals (iRFP channel) by confocal microscopy. The nucleus was counterstained with DAPI (blue channel). Scale bar, 50 μm. (**C**) Reciprocal co-IP to assess interactions between the nascent NS3 and endogenous QPRT after Huh7.5.1 cells were infected with HCV for 48 h. Mouse IgG was used as a negative control. (**D**) Immunoblotting of HA-QPRT cleavage in the presence of co-expressed Flag-NS3/4A in 293T cells for 24 h. Reduction of QPRT (top panel) and lack of cleaved fragments of QPRT (middle panel, long exposure). (**E**) Ubiquitination of myc-QPRT in the presence of HA-Ubi was assessed by co-IP after Huh7.5.1 cells were transiently co-transfected with Flag-NS3/4A, or infected with HCV, for 24 h. IP efficiency was measured by immunoblotting with Myc antibody, and HCV replication was assessed with NS3 antibody in WCL. (**F**) Degradation of endogenous QPRT was assessed by immunoblotting after Huh7.5.1 cells were infected with HCV for 48 h, in the absence or presence of MG132 treatment for additional 12 h. (**G**) Degradation of Myc-QPRT was monitored by immunoblotting with co-transfected E3 ligases, Flag-Smurf2, V5-TRIM25 or Flag-HOIP, in Huh7.5.1 cells for 48 h. (**H**) co-IP to measure interaction between Myc-QPRT with NS3/4A in the presence of co-transfected mock or different Smurf2 siRNA after HCV infection of Huh7.5.1 cells for 48 h. (**I**) co-IP analysis of the co-expressed Flag-Smurf2 with HA-NS3 and Myc-QPRT in Huh7.5.1 cells. Mouse IgG was used as a negative control. (**J**) Decay of Myc-QPRT and different mutants as indicated was compared by immunoblotting with or without co-transfected Flag-Smurf2 in Huh7.5.1 cells. Full-length gels and blots are included in the Figures S6 and S7.

HCV NS3/4A can direct cleave the type I interferon signaling adaptors, TRIF and MAVS, to evade the innate immune surveillance^20^. To investigate whether a decreased level of QPRT was possibly caused by NS3/4A cleavage, we first performed co-immunoprecipitation (co-IP), which indicated that overexpressed HA-QPRT and Flag-NS3 in HEK293T cells formed a complex (Fig. 2A). The interaction between QPRT and NS3/4A was further confirmed by far-red fluorescence complementation (BiFC) analysis^21^, where HeLa cells co-expressed with QPRT-iRN97 and iRC98-NS3/4A were specifically lit up (Fig. 2B). Reciprocal co-IP (Fig. 2C) and confocal microscopy (Figure S2A) analyses further confirmed that the endogenous QPRT interacted with the nascent NS3 and co-localized in the cytoplasm after HCV infection in Huh7.1.5 cells. Therefore, these results indicated that QPRT could make direct protein-protein contact with HCV NS3/4A.

Such a close proximity would allow NS3/4A to cleave QPRT. Unexpectedly, however, HA-NS3/4A failed to cleave the co-expressed QPRT in 293T cells (Fig. 2D), despite of its effective cleavage of VISA/MAVS (Figure S2B). Therefore, reduction of QPRT protein levels would not require NS3/4A protease activity. In search for other potential mechanism, we noticed that QPRT ubiquitinylation was evident in response to HCV infection or in the presence of the co-expressed NS3/4A in Huh7.5.1 cells, in a dose dependent manner (Fig. 2E). Addition of the proteasome inhibitor, MG132, to Huh7.5.1 cells attenuated HCV-induced QPRT degradation (Fig. 2F) and increased the accumulation of ubiquitination of QPRT by the overexpressed FLAG-NS3/4A (Figure S2C). A faster decay of QPRT in response to HCV infection after cycloheximide (CHX) inhibition of protein *de novo* synthesis also indicated that post-translational modification was involved in QPRT down-regulation (Figure S2D and E). Therefore, these results suggested that HCV NS3/4A could promote QPRT degradation through the proteasomal pathway. A variety of E3 ubiquitin ligases, Smurf2, Trim25 or HOIP, have been suggested to regulate HCV infection^22–24^. After each E3 ligase was co-expressed with QPRT in Huh7.5.1 cells, only ectopically expressed Smurf2, but not TRIM25 or HOIP, could enhance QPRT protein degradation in a dose dependent manner (Fig. 2G). Of note, Smurf2 expression *per se* was upregulated by HCVcc infection (Figure S2F). Moreover, down-regulation of Smurf2 expression by siRNA in Huh7.5.1 (Figure S2G and S2H for RNAi efficacy) could effectively attenuate the interaction between NS3/4A and QPRT (NS3 panel of co-IP), and reduce HCV-mediated QPRT decay (myc panel of WCL) (Fig. 2H). Furthermore, co-IP experiment showed that FLAG-Smurf2 could pull down both HA-tagged NS3/4A and myc-tagged QPRT (Fig. 2I), indicative of formation of a functional tripartite complex. Thus, Smurf2 would likely involve in inter-molecular interaction between NS3/4A and QPRT, and promoting QPRT proteasomal degradation by HCV infection. To identify the potential ubiquitin conjugation sites within QPRT, Alanine substitution to K139, K171 and K288 was carried out. Mutant derivatives of QPRT became resistant to proteolysis in the presence of co-expressed Smurf2 in Huh7.5.1 cells (Fig. 2J). In conclusion, HCV infection likely targeted Smurf2 mediated proteasomal degradation of QPRT.

### The enzymatic activity of QPRT was required to suppress HCV replication

**Figure 3:**
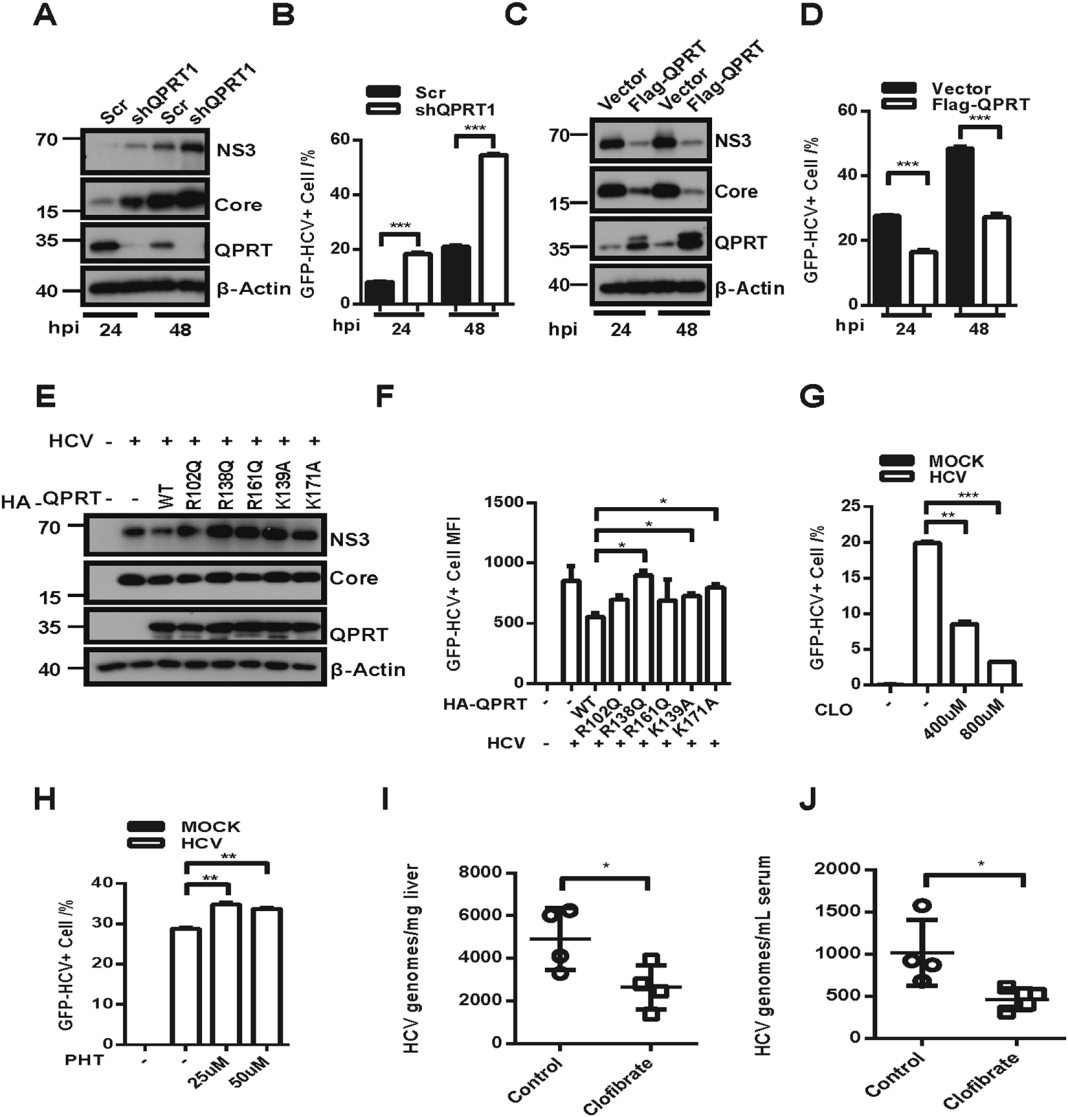
QPRT suppressed HCV replication dependent on its enzyme activity. (**A–D**) Huh7.5.1 cells stably expressing QPRT shRNA (shQPRT#1) or Flag-QPRT (Flag-QPRT) were infected with HCV for indicated time. (**A,C**) HCV replication was assessed by immunoblotting of NS3 and Core, and (**B,D**) percentage of GFP^+^ cells in FACS. Expression of QPRT and actin loading control were shown. (**E,F**) HCV replication was assessed as in (**A–B**) after Huh7.5.1 cells were transfected with HA-QPRT or the indicated enzyme-dead mutants for 6 h. Expression of QPRT and β-actin loading control were shown. (**G–H**) After Huh7.5.1 cells were infected with HCV for 6 h, (**G**) QPRT agonist (CLO) or (**H**) QPRT inhibitor (PHT) was added for 48 h at the indicated doses. HCV replication was quantified by proportion of GFP^+^ cells. (**I–J**) C/O^Tg^ mice (6–8 weeks old) were infected with 1 mL of HCV J399EM (TCID_50_ = 1 × 10^8^/mL) for 3 wks, then treated with CLO (8 mg in 100 μL of corn oil, daily, *i.p*.) for 2 wks. HCV RNA copies in livers (**I**) or sera (**J**) were measured by qRT-PCR. Full-length gels and blots are included in the Figure S8. Error bars were SEM of three independent experiments. Student *t* test, *P < 0.05; **P < 0.01, ***P < 0.01.

To further determine whether QPRT is required to control HCV replication, we downregulated the expression of QPRT by stably expressed shRNA in Huh7.5.1 cells (Figure S3A for knockdown efficiency). Less QPRT led to a more efficient HCV replication, as indicated by the increased expression of HCV proteins (Fig. 3A) and viral genome replication (Fig. 3B), compared to the mock treatments. QPRT deficiency also associated with the elevated HCV assembly (Figure S3B), budding efficiency (Figure S3C), and the specific infectivity (Figure S3D). In contrast, stable overexpression of Flag-QPRT in Huh7.5.1 cells led to significant reduction of HCV Core and NS3 proteins (Fig. 3C) and the viral replication efficiency (Fig. 3D), compared to the vector controls. Intracellular (Figure S3E) and extracellular (Figure S3F) HCV titers were also significantly decreased in QPRT overexpressed cells (Figure S3E-F). These results therefore indicated that QPRT was a potential host factor resisting to HCV replication.

The enzymatic active site of QPRT for QA binding^25^ consists of three Arginine (R102, R138 and R161) and two Lysine residues (K139 and K171). To investigate whether the catalytic activity of QPRT would involve in inhibition of HCV replication, each of these basic residues was mutagenized and transiently overexpressed in Huh7.5.1 cells. Loss-of-function mutants in general could no longer attenuate HCV replication, with R138Q, K139A and K171A mutation exhibiting the most pronounced effect (Fig. 3E,F). To substantiate that QPRT relied on its enzymatic activity to suppress HCV replication in cells, HCV replication in Huh7.5.1 cells was further measured in the presence of a QPRT agonist, clofibrate (CLO), or an antagonist, phthalic acid (PHT). To our expectation, CLO (Fig. 3G) could reduce and PHT (Fig. 3H) enhance HCV replication, respectively. The inhibitive effect of CLO on HCV replication at least partly relied on QPRT, since down regulation of QPRT made CLO less effective to inhibit HCV replication (Figure S3G and H). More significantly, QPRT agonist could inhibit HCV replication *in vivo*. When CLO was administrated (8 mg, *i.p*, daily for 2 weeks) in C/O^Tg^ mice two weeks after HCV had established persistent infection, the viral loads in liver (Fig. 3I) and serum (Fig. 3J) were reduced by 2-fold. Therefore, these results suggested that QPRT would involve in cytosolic control of HCV replication, and activation of its catalytic activities might provide a therapeutic benefit against chronic hepatitis C.

### QPRT inhibited HCV replication via reduction of cellular lipogenesis

**Figure 4:**
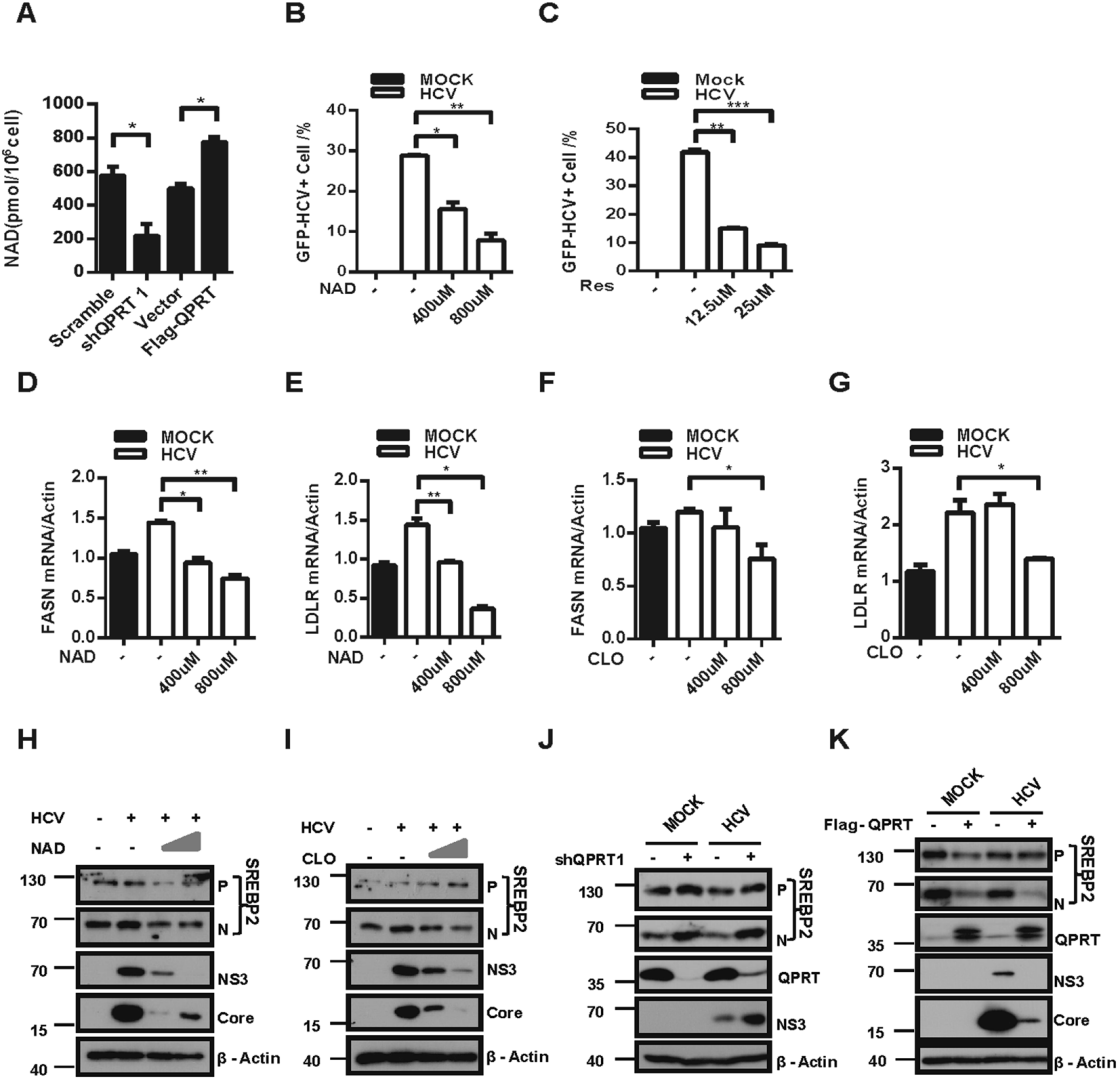
QPRT inhibited HCV replication through modulation of NAD-dependent lipogenesis. (**A**) The intracellular NAD levels were colorimetrically measured in shQPRT cells and Flag-QPRT overexpressing cells. Huh7.5.1 cells were infected with HCV for 6 h, followed by treatments of NAD (**B, D–E,H**), Res (**C**) or CLO (**F,G,I**) for 48 h at indicated doses. HCV replication was measured by FACS (**B,C**), mRNA levels of FASN (**D,F**) and LDLR (**E,G**) were measured by real-time PCR. (**H,I**) Precursor (**P**) and active nuclear forms (**N**) of SREBP2, NS3, Core and β-actin loading control were detected by immunoblotting. (**J,K**) shQPRT cells or Flag-QPRT cells were infected with HCV for 48 h. SREBP2, NS3, Core, QPRT and β-actin loading control were detected by immunoblotting. Full-length gels and blots are included in the Figure S9. Error bars were SEM of three independent experiments. Student *t* test, *P < 0.05; **P < 0.01, ***P < 0.01.

QPRT is primarily responsible for *de novo* synthesis of NAD. Indeed, the intracellular NAD concentrations effectively responded to the varying protein levels of QPRT, by either RNAi or overexpression, in Huh7.5.1 cells (Fig. 4A). Further, addition of cell permeable NAD^26^ to Huh7.5.1 cells could inhibit HCV replication in a dose dependent manner (Fig. 4B). Sirt1 is a NAD^+^-dependent deacetylase^11^, whose activity and expression are both required to inhibit lipid droplets-dependent HCV replication in hepatocytes^12, 13^. In agreement to these findings, addition of Sirt1 activator Resveratrol (Res) inhibited HCVcc replication in Huh7.5.1 cells (Fig. 4C), as effectively as that by NAD (Fig. 4B). Resveratrol can activate Sirt1 to inhibit lipid droplets synthesis, the latter is essential for HCV assembly step as evidenced in Fig. 4C. This was contradictory to a previous report using HCV replicon cells^27^, where the sub-genomic HCV replication does not require the assembly step. Therefore, activation of QPRT or increase of its catabolite NAD would benefit an antiviral effect, potentially through inactivation of cellular lipogenesis.

To substantiate this notion, qPCR analyses showed that NAD or CLO treatment of cells caused a significant decrease of fatty acid synthase (FASN; Fig. 4D,F) and low density lipoprotein receptor (LDLR; Fig. 4E,G), two lipogenic genes required for efficient HCV replication. The inhibitive potency of NAD or CLO on FASN expression was similar to that by overexpressed QPRT in Huh7.5.1 cells (Figure S4A). Consistently, QPRT knockdown in Huh7.5.1 cells caused an increase of FASN (Figure S4B) and LDLR (Figure S4C) expression. Furthermore, NAD activation of Sirt1 can deacetylate thus destabilize SREBP-1/2, the transcription activators of FASN and LDLR gene expression^28, 29^. In fact, HCV infection or QPRT knockdown in Huh7.5.1 cells could activate SREBP, as indicated by the increase of the nuclear active form of SREBP2 (Fig. 4H–J). NAD or CLO treatment and overexpression of QPRT, on the other hand, reduced the nuclear translocation of SREBP2 (Fig. 4H,I,K), indicative of the involvement of QPRT on modulation of Sirt1-SREBP pathway. Therefore, QPRT might inhibit HCV replication through the control of NAD/Sirt1 lipogenesis required for efficient viral replication and infection.

## Discussion

The previous reports have shown that IFNγ can up-regulate QPRT in human peripheral blood mononuclear cells^30^, and promote *de novo* NAD synthesis in murine macrophages cell line^31^, suggesting the potential involvement of QPRT in innate immunity. This work has shown that HCV likely can activate Smurf2-mediated proteasomal degradation of QPRT for a more efficient viral replication. One possible mechanism is that the reduction of QPRT inevitably decelerates *de novo* NAD synthesis and accelerate the lipogenesis, the latter would favor an efficient HCV replication. Therefore, QPRT may provide a cytosolic immunity against HCV replication through affecting NAD/Sirt1 pathway.

The potential anti-HCV role of NAD has been suggested previously by rather sparse reports^12, 13^, but exactly how NAD regulates HCV replication remains unknown. We show in this work that the reduced NAD/NADH ratio in hepatocytes after HCV infection may be caused by a reduced QPRT catabolism. We have further provided evidence, both *in vitro* and *in vivo*, that modulation of enzymatic activities of QPRT, by its activator or inhibitor, can effectively modulate HCV replication efficiency. In consequence, intracellular NAD would reflect both the amount and activity of QPRT, and execute its potential anti-HCV activity through Sirt1. We show in this study that increased NAD or activation of QPRT by CLO both can inactivate SREBP2 and downregulate expression of FASN and LDLR, two essential lipogenic genes required for HCV infection.

In sum, we tentatively conclude that QPRT involve in cytosolic immunity against HCV replication through insurance of an unfavorable NAD/Sirt1 lipogenesis. Reduced QPRT activity, thus impaired QPRT immunity occurred in chronic hepatitis C, can be reconstituted by QPRT agonist. This also point to a potential therapeutic target for hepatitis C. Of course, one needs to develop better QPRT agonists than CLO in this respect. This is not only because the relatively lower potency of CLO in reduction of HCV titers, but also because CLO, a lipid lowering drug, can reduce serum cholesterol and converts LDL to HDL. Higher serum cholesterol and LDL levels have been suggested to increase the probability of a sustained viral response (SVR) by IFN based therapy in chronic hepatitis C^32^. The situation in using CLO is further complicated that CLO is also a PPARα activator, and long-term treatment of HCV core-transgenic mice with CLO may induce hepatocarcinogenesis^33^. Despite of all these complications associated with CLO, this work shed a light onto development of a more specific and potent QPRT enzymatic activator, that may help stockpile more arsenals in the endeavor of HCV cure in the future.

## Materials and Methods

### Animals and Patients

The humanized mice (C/O^Tg^) permissive for HCV were used for HCV infection, exactly as previously described^19^. In brief, C/O^Tg^ (n = 5, male, 8–12 weeks of age) were tail vein inoculated with HCV J399EM (TCID_50_ = 1 × 10^8^/mL) or mock saline. Mice were randomly withdrawn at the indicated time points and sacrificed for analyses of viral loads and QPRT expression. Human liver tissues were collected using biopsy needles guided by ultrasound scan or during a laparoscopy. Tissues were divided into three groups, health volunteer (HV), chronic hepatitis C (CHC) and cirrhosis with chronic HCV infection, and four groups with disease grade (G1, G2, G3 and G4) of hepatitis patients as previously described^34^ (Table S1).

### Reagents and Antibodies

Mouse monoclonal antibodies against FLAG (Sigma, F1804), Myc (CST, 2276S), HCV NS3 (Abcam, ab65407) and Core (Thermo, MA1-080), rabbit monoclonal antibody against HA (CST, 3724S), QPRT (Abcam, ab180930), Smurf2 (CST, 12024), rabbit polyclonal antibody against SREBP-2 (Santa Cruz, sc-13552), and fine chemicals of proteasome inhibitor MG132 (ApexBio Tech, A2585), 2,3-Pyridinedicarboxylic acid (QA, Sigma, P63204), 2,3-Pyridinedicarboxylic Acid-d3 (QA-d3, J&K Scientific Ltd, P991633), β-Nicotinamide adenine dinucleotide hydrate (NAD, Sigma, N7004), Resveratrol (Res, Sigma, V900386), Phthalic acid (PHT, Sigma, 80010), Clofibrate (Sigma, C6643) and cycloheximide (Sigma, C7698) were purchased from where indicated.

### Expression Vectors, tissue cultures, virus stocks

The full length human QPRT was amplified by RT-PCR and cloned into various expression vectors where indicated: pCMV-HA, pCMV-myc, pcDNA3.1-N-Flag (courtesy of Prof. Dacheng Wang, Institute of Biophysics, CAS). Site-directed mutagenesis (TransGen Biotech) was performed to generate enzyme inactive mutants of QPRT (R102Q, R138Q, R161Q, K139A and K171A), based on pCMV-HA-QPRT, and potential ubiquitin conjugation site mutants of QPRT (K139A, K183A and K288A), based on pCMV-Myc-QPRT. The expression vectors of HCV NS3/4A, and pXJ40-Flag-NS3/4A were described previously^35^. pCMV-HA-Ubi expression vector (Dr. Yanyi. Wang, Wuhan Institute of Virology, CAS), Flag-Smurf2 expression vector (Dr. Jun Zhang, Peking University Health Science Center), V5-TRIM25 expression vector (Dr. Zhaocai Zhou, Shanghai Institute of Biochemistry and Cell Biology, CAS) and Flag-HOIP expression vector (Dr. Hongyu Deng, Institute of Biophysics, CAS) were kindly provided as indicated. HCV NS3/4A coding sequence was sub-cloned from pJFH1(Dr. Takaji Wakita, National Institute of Infectious Diseases, Japan) into pCDNA3.1-bFos-iRC98, and QPRT into pcDNA3.1-bJun-iRN97, to replace Jun or Fos, respectively, for BiFC assays (Dr. Zongqiang Cui, Wuhan Institute of Virology, CAS). The coding sequences of short hairpin RNAs (shRNA. shQPRT#1, [5′-GCCTTTCTTCGATGCCATATT-3′]; shQPRT#2 [5′-GTGGCAGGCACGAGG AAGA-3′]; shQPRT#3 [5′-GATGGTG AAGGATAACCATGT-3′]) targeting the QPRT gene were cloned into shRNA expression vector psi-mU6 (Genecopoeia). The siRNA sequences (siSMURF2 #746, [5′-CGGGCCAAATGACAATGAT-3′]; siSMURF2 #831, [5′-GTGGACTGCAGTC GTTTAT-3′]; siSMURF2 #1405, [5′-GGCAGAACCAATTGAAAGA-3′]; and the scramble, [5′-TTCTCCGAACGTG TCACGT-3′]) targeting the Smurf2 gene were ordered from GenePharma (China).

The HCV infectious clone J399EM and the measurement of 50% tissue culture infective dose (TCID_50_) have been described previously^36^. Virus titers in cell lysates and supernatants were detected for TCID50 by endpoint dilution assays (EPDA) as previously described^37^. MOI = 0.1 was routinely applied to infect Huh7.5.1 cells (courtesy of Dr. Jin Zhong, Institute Pasteur of Shanghai, CAS). Cells were routinely cultured in DMEM (Invitrogen) supplemented with 2 mM L-glutamine, 2 mM nonessential amino acids, 100 mM HEPES and 10% FBS (Invitrogen, 10099-141). Huh7.5.1 cells stably expressing QPRT shRNA were selected and maintained in the presence of 2 μg/mL Puromycin (Sigma, P8833), and those stably expressing Flag-QPRT with 800 μg/mL G418 (Millipore, 345810).

### Flow cytometry

To quantify the kinetics of viral replication, Huh7.5.1 cells infected with HCV J399EM for the indicated time, trypsinized and fixed in 4% paraformaldehyde before subject to flow cytometric analysis in FACSAria III (BD Biosciences, CA). GFP channel excited at 488 nm was acquired and analyzed using FlowJo7.6.1 software (Ashland, OR).

### Confocal microscopy

BiFC was used to assess protein-protein interaction as previously reported^21^. In brief, HeLa cells (2 × 10^5^) were transfected with pcDNA3.1-NS3/4A-iRC98 and pCDNA3.1-QPRT-iRN97 (a total of 1 μg) for 48 h. After paraformaldehyde fixation, cells were incubated in 1 µg/mL Hoechst3342 (DNA stain) for 1 min before mounting. Red fluorescence from the iRFP channel (λ_ex_ = 640 nm), and the nuclei counterstained with Hoechst 33342 (λ_ex_ = 405 nm) were captured using a UltraView VOX confocal system (Perkin Elmer), and analyzed with Volocity software (Perkin Elmer).

### Quantitative PCR

Total RNA was extracted from liver (0.1 g), cells (1 × 10^6^), sera (0.1 mL), or cell culture medium (250 μL) using Trizol or Trizol LS reagent (Invitrogen). qPCR reactions were performed as described previously^38^ in an StepOne Plus (ABI, CA), using iTaq™ Universal SYBR® Green Supermix (Bio-Rad). Primers for indicated genes were listed in Table S2. HCV RNA copy numbers in liver tissues, sera, cell lysates and culture media were measured as previously described^19^. Data were analyzed with ABI software version 2.0.3 (Applied Biosystems).

### Immunoprecipitation and Immunoblotting

Experiments were performed as described previously^39^. In brief, 293T cells (5 × 10^5^) were transfected with indicated expression plasmids (2 μg) by jetPRIME reagents (114-07, Polyplus S.A.) for 24 h. Cell lysates (400 μL) were immunoprecipitated by indicated antibody (1 μg) using Prorein G Dynabeads (Life Technology). Protein complexes were further resolved in SDS-PAGE, and detected by immunoblotting with the primary antibodies where indicated. Chemiluminescent HRP Substrate (Millipore) was used to detect indicated bands.

### Immunohistochemistry

IHC staining of various patient liver tissues was performed as previously described^19^, except that the rabbit monoclonal antibody against QPRT (1:200, Abcam) was used as the primary antibody. Images were acquired by Digital Pathology system (Pannoramic MIDI, Hungary) and analyzed with Pannoramic Viewer software (3dHISTECH, Hungary).

### Measurement of intracellular NAD and quinolinic acid

After cells (2 × 10^5^) where indicated were washed with cold PBS three times, sample preparation and intracellular NAD measurement were carried out as described in specification of NAD/NADH Quantification Colorimetric Kit (BioVision). The sample preparation^40^ for LC-MS analysis^41^ of quinolinic acid (QA) was performed as previously described. Briefly, 10 μM deuterium D3 quinolinic acid (QA-d3, J&K Scientific Ltd) was spiked to the culture supernatants as the internal standard. Proteins in supernatants were precipitated by trichloracetic acid (Sigma, 3% final concentration), the supernatant was then filtered through a spin column (MW cutoff = 3 kDa, Millipore Amicon) at 14,000 × g at 4 °C for 10 min. The filtrates were 10 × diluted and injected (5 µL) to a Acquity H-Class UPLC system (Waters, HSS T3 2.1 × 100 mm, 1.8 µm particle column. Solution A, 0.3% formic acid in water, and solution B, 0.3% methanol in water). The analytes were eluted (0.3 mL/min flow rate) by the following gradients: 0–1.7 min (95%A and 5%B), 5.0–6.0 min (50% A and 50%B) and 6.5–9.0 min (95%A–95%B). The triple quadrupole in the + ve ESI mode was used in a Quattro Premier XE mass spectrometer (Waters, MA). Standard solutions of QA for calibration curves were prepared from stock solutions. The final concentrations of unknowns were calculated by interpolation of the standard curves.

### Protein turnover assay

The half life of QPRT protein was assessed by cycloheximide (CHX) chase assay as described previously^42, 43^. In brief, Huh7.5.1 cells were infected with HCVcc for 48 h, 300 μg/mL CHX was then added to inhibit *de novo* protein synthesis. At the indicated time, cells lysates were prepared for immunoblotting of QPRT and HCV NS3. After linear regression of QPRT levels, the half life of QPRT was calculated from the slope of the best fit line.

### Statistical analysis

Data were analyzed using unpaired two-tailed student’s *t*-test with GraphPad Prism 6 software (San Diego, CA). *P* values < 0.05 were considered statistically significant.

### Ethics Statement

Animal experiments were performed in accordance with the National Institutes of Health guidelines and were approved by the Animal Care and Use Committee, Institute of Biophysics, Chinese Academy of Sciences. Clinical samples were collected and studied according to the experimental practices and standards approved by the Medical Ethics Committee of First Hospital of Jilin University (approval code: 2015–235), and informed consent was signed by patients enrolled in the study.

## Electronic supplementary material

Supplementary Information
