# Supplementary material for: Quinolinate Phosphoribosyltransferase is an Antiviral Host Factor Against Hepatitis C Virus Infection
Source: Sci Rep. 2017 Jul 19;7:5876. doi: 10.1038/s41598-017-06254-4 (PMC5517448; doi:10.1038/s41598-017-06254-4)
Supplement: Supplementary file 1 — Supplementary Information [file 41598_2017_6254_MOESM1_ESM.pdf]

# Quinolinate Phosphoribosyltransferase is an Antiviral Host Factor Against Hepatitis C Virus Infection

Zhilong Wang[1a, 2, 6 +], Yanhang Gao[5,+], Chao Zhang[2], Haiming Hu[1a], Dongwei Guo[4], Yi Xu[1b], Qiuping Xu[1a, 3], Weihong Zhang[1a], Sisi Deng[1a], Pingyun Lv[1a], Yan Yang[1a], Yanhua Ding[5], Qingquan Li[5], Changjiang Weng[4], Xinwen Chen[1a], Sitang Gong[1b], Hairong Chen[2], Junqi Niu[5] and Hong Tang[1a,2,3,*]

[1] The Joint Center for Translational Precision Medicine, [a] Wuhan Institute of Virology, Chinese Academy of Sciences, Hubei, China 430071 and [b] Guangzhou Women and Children's Medical Center, Guangzhou, China 510623

[2] CAS Key Laboratory of Infection and Immunity, Institute of Biophysics, Chinese Academy of Sciences, Beijing, China 100101

[3] Institut Pasteur of Shanghai, Chinese Academy of Sciences, Shanghai, China 200031

[4] Harbin Veterinary Research Institute, Chinese Academy of Agricultural Sciences, Heilong Jiang, China 150001

[5] Department of Hepatology, The First Hospital of Jilin University, Changchun, Jilin, China 130021

[6] University of Chinese Academy of Sciences, Beijing, China 10049

[*] Correspondence should be addressed to H.T. email：htang@ips.ac.cn

+ These authors contributed equally to this work

## Supplementary Figure Legends

Figure S1. Measurement of HCV RNA genomic copies in patient serum, C/O$^{Tg}$ hepatocytes and Huh7.5.1 cells, related to Fig 1.

(A) Serum HCV RNA in chronic hepatitis C patients used in Fig 1A and 1B was qPCR measured. Pearson's correlation analysis was performed on HCV RNA genome copies and the scores of QPRT positivity in liver biopsies at different disease grades. R denotes Pearson's correlation coefficient. (B) C/O$^{Tg}$ mice were infected with HCV as described in Fig. 1C. Liver HCV genome copies were measured by qPCR at the indicated time post HCV inoculation. (C) Huh7.5.1 cells were infected with HCV J339EM (MOI = 0.1, 0.2 and 0.4) for 48 h. HCV RNA copies were measured by qPCR, and the percentage of GFP + cells was quantified in FACS. The correlation between HCV RNA genomic copies and the percentage of GFP + cells were plotted. Error bars were SEM of three independent experiments, student *t* test, **P*<0.05; ***P*<0.01, ****P*<0.001.

Figure S2. HCV NS3 triggered Smurf2 mediated proteasomal degradation of QPRT, related to Fig 2.

(A) Huh7.5.1 cells were infected with HCV J339EM (MOI = 0.1) for 48 h. Co-localization between endogenous QPRT (green) and NS3/4A (red) in Huh7.5.1 cells were assessed by confocal microscopy. The nuclei were counterstained with DAPI (blue). White arrows in merged panels indicated the co-localization signals. Scale bar, 50 μm. (B) Flag-NS3/4A and HA-VISA were co-expressed as described in Fig. 2D and the proteolytic products were marked by asterisk. (C) Ubiquitination of myc-QPRT in the presence of HA-Ubi was assessed by co-IP after Huh7.5.1 cells were transiently co-transfected with Flag-NS3/4A for 12 h and treated in the absence or presence of MG132 for additional 12 h. The IP efficiency was assessed by immunoblotting with Myc antibody, and Flag-NS3/4A was assessed with Flag antibody in WCL. (D) The decay of QPRT in HCV infected Huh7.5.1 cells. Two days after mock or HCVcc infection, Huh7.5.1 cells were spiked with CHX for 30 min. Immunoblotting of QPRT and NS3 was carried out after cells were further cultured for the indicated time. (E) Relative amounts of QPRT normalized to actin were plotted and the decay slopes represented the turnover rates. (F) Smurf2 mRNA levels were measured by qPCR after Huh7.5.1 cells were infected with HCV for 48 h. (G-H) Huh7.5.1 cells were transfected with the indicated siRNA against Smurf2 for 48 h. The levels of (G) Smurf2 mRNA and (H) protein were detected by qPCR or immunoblotting, respectively. Full-length gels and blots are included in the Figure S10.

Figure S3. Down regulation of QPRT increased HCV replication and alleviated the inhibitive effect of CLO on HCV replication. Related to Fig. 3.

Huh7.5.1 cells stably expressing QPRT shRNA (shQPRT #1 to 3) were infected with HCV (MOI = 0.1) for 48 h. (A) Indicated proteins were detected by immunoblotting. Intracellular (intra) and supernatant (sup) Both HCV RNA copies and viral titers in scrambled shRNA or shQPRT#1 treated cells were measured by qPCR and endpoint dilution assays, respectively. (B) The assembly efficiency was plotted as the ratios of HCV RNA copies in sup over intra (sup/intra), (C) budding efficiency as ratios of titers in sup over intra (sup/intra), and (D) specific infectivity as the ratios of sup HCV titer/sup HCV copies in log scales. Huh7.5.1 cells transiently expressing HA-QPRT were infected with HCVcc (MOI = 0.1) for 48 h. (E) Intracellular (intra) and (F) supernatant (sup) HCV titers were measured by EPDA. Huh7.5.1 cells stably expressing QPRT shRNA #1 were infected with HCV (MOI = 0.1) in the absence or presence of QPRT agonist (CLO) for 48 h. (G) QPRT, NS3 and Core were detected by immunoblotting, and (H) HCV replication was quantified by FACS as the percentage of GFP + cells. Full-length gels and blots are included in the Figure S11

Figure S4. QPRT inhibited SREBPs target gene expression in Huh7.5.1 cells, related to Fig. 4. Huh7.5.1 cells with (A) Flag-QPRT stably overexpressed or (B-C) QPRT stably knocked down were infected with HCV J399EM for 48 h. mRNA levels of FASN (A-B) and LDLR (C) were measured by qPCR, and ratios of copy numbers to β-actin were plotted.

Figure S5. Full-length gels and blots, related to Fig. 1.

Figure S6. Full-length gels and blots, related to Fig. 2(A, C, D, E).

Figure S7. Full-length gels and blots, related to Fig. 2((F, G, H, I, J).

Figure S8. Full-length gels and blots, related to Fig. 3.

Figure S9. Full-length gels and blots, related to Fig. 4.

Figure S10. Full-length gels and blots, related to Figure S2.

Figure S11. Full-length gels and blots, related to Figure S3.

Figure S1

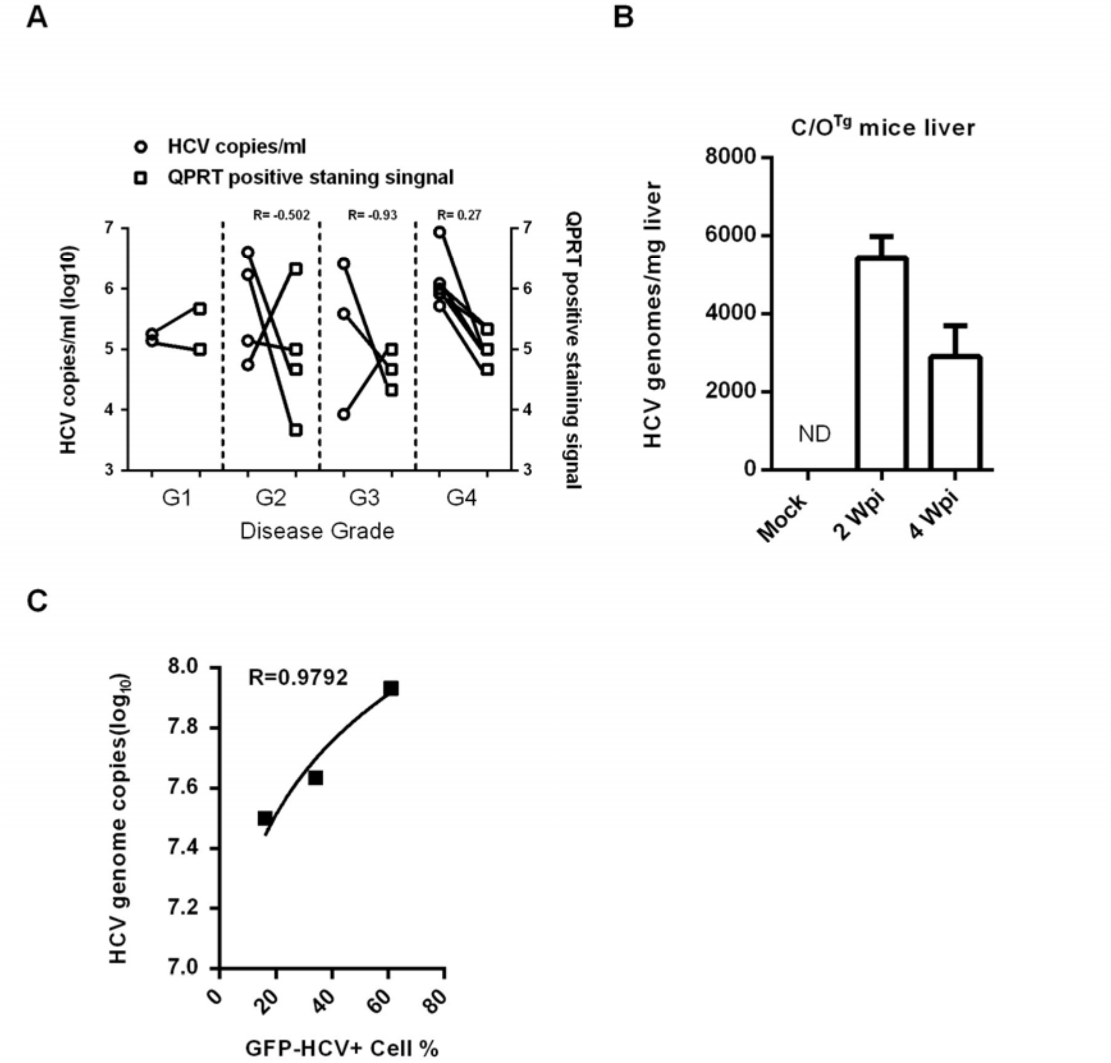
A
HCV copies/ml
QPRT positive staning singnal
R= -0.502
R= -0.93
R= 0.27
HCV copies/ml (log10)
QPRT positive staining signal
G1
G2
G3
G4
Disease Grade
B
C/O^Tg mice liver
HCV genomes/mg liver
ND
Mock
2 Wpi
4 Wpi
C
R=0.9792
HCV genome copies(log10)
GFP-HCV+ Cell %

Figure S2

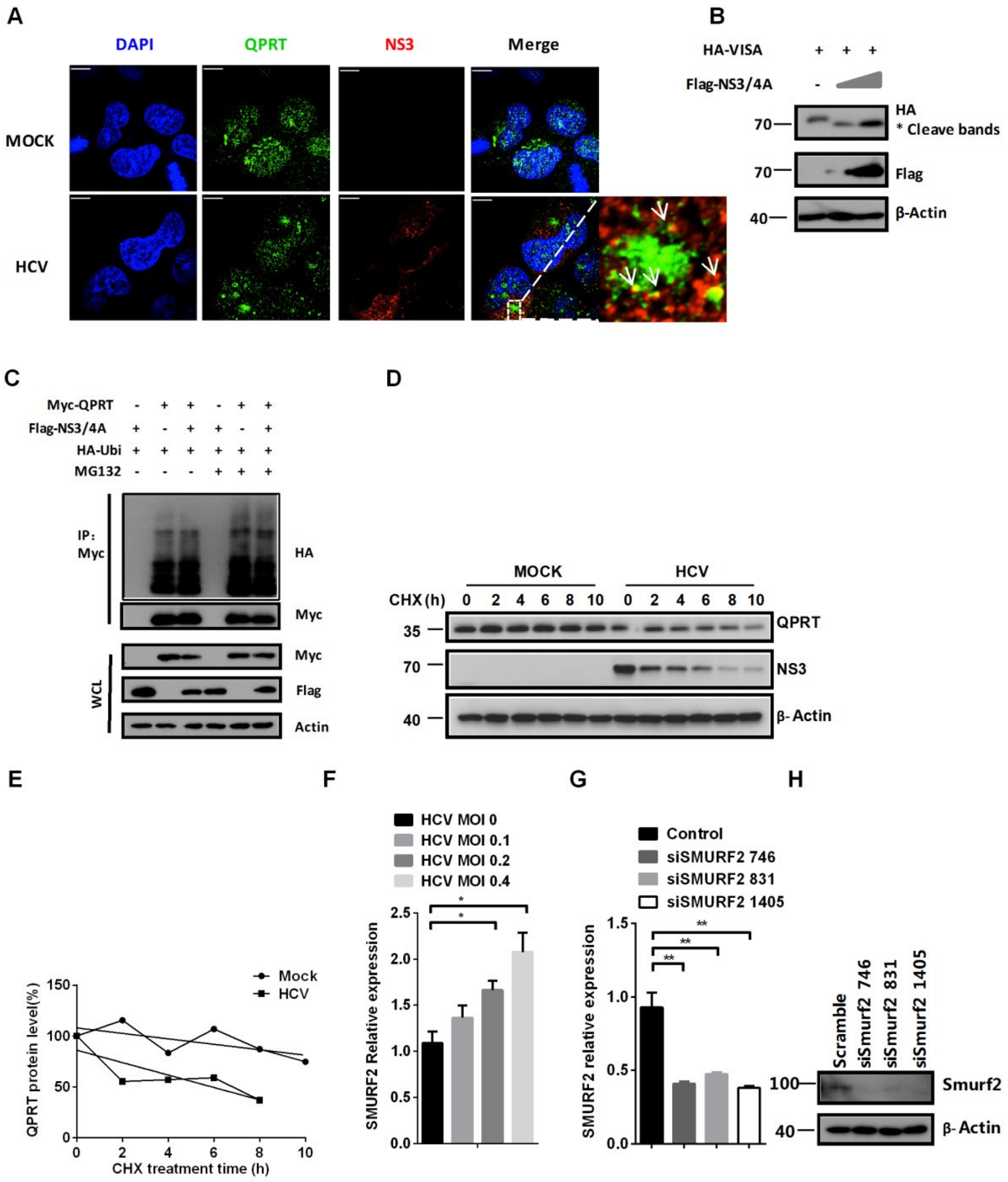
A
DAPI
QPRT
NS3
Merge
MOCK
HCV
B
HA-VISA + + +
Flag-NS3/4A -
70
HA
* Cleave bands
70
Flag
40
β-Actin
C
Myc-QPRT - + + - + +
Flag-NS3/4A + - + + - +
HA-Ubi + + + + + +
MG132 - - - + + +
IP: Myc
HA
Myc
WCL
Myc
Flag
Actin
D
MOCK
HCV
CHX (h) 0 2 4 6 8 10 0 2 4 6 8 10
35
QPRT
70
NS3
40
β- Actin
E
Mock
HCV
QPRT protein level(%)
CHX treatment time (h)
F
HCV MOI 0
HCV MOI 0.1
HCV MOI 0.2
HCV MOI 0.4
SMURF2 Relative expression
G
Control
siSMURF2 746
siSMURF2 831
siSMURF2 1405
SMURF2 relative expression
H
Scramble
siSmurf2 746
siSmurf2 831
siSmurf2 1405
100
Smurf2
40
β- Actin

Figure S3

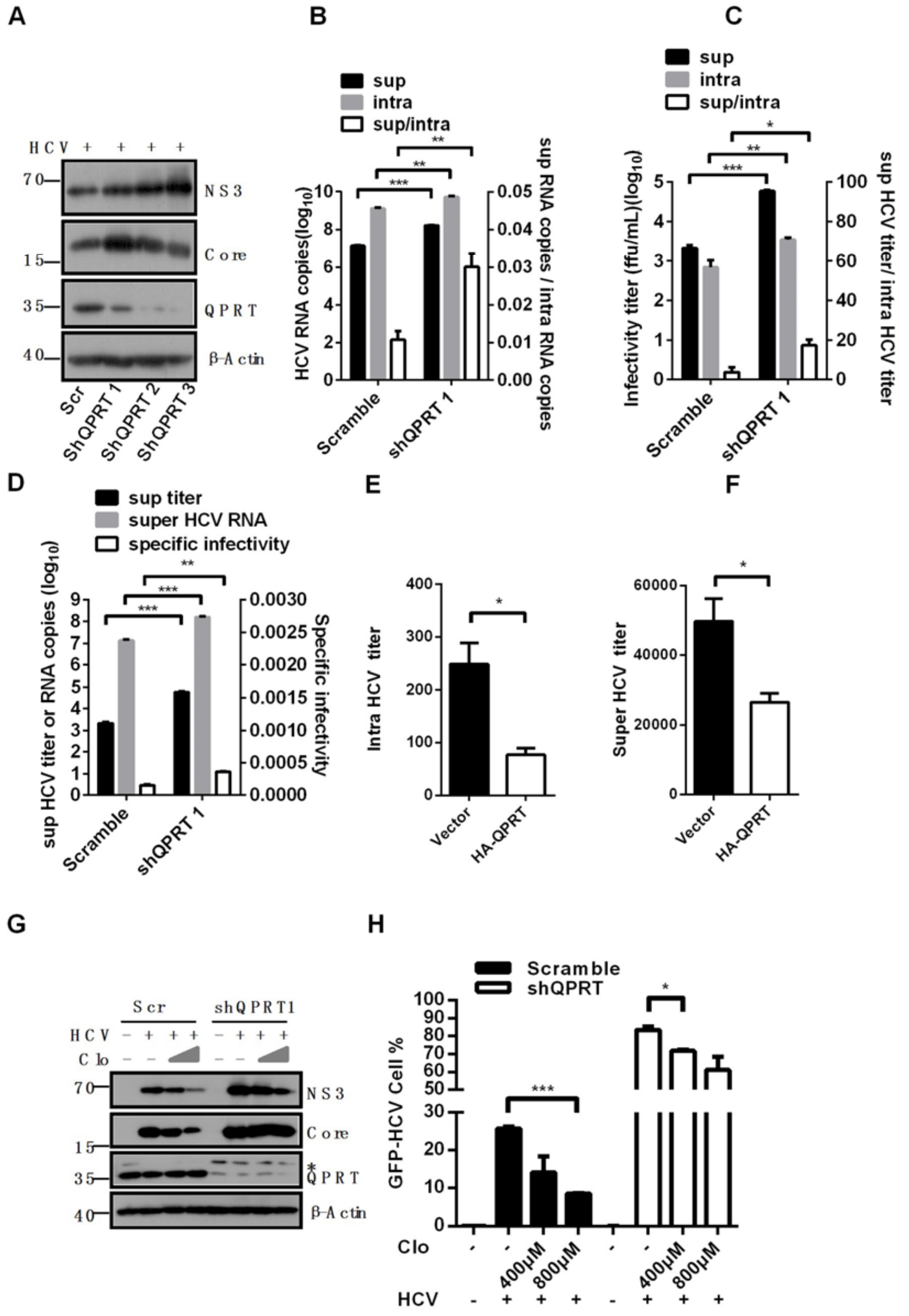
A
HCV + + + +
70
15
35
40
NS3
Core
QPRT
β-Actin
Scr
ShQPRT 1
ShQPRT 2
ShQPRT 3
B
sup
intra
sup/intra
HCV RNA copies(log10)
sup RNA copies / intra RNA copies
Scramble
shQPRT 1
C
sup
intra
sup/intra
Infectivity titer (ffu/mL)(log10)
sup HCV titer/ intra HCV titer
Scramble
shQPRT 1
D
sup titer
super HCV RNA
specific infectivity
sup HCV titer or RNA copies (log10)
Specific infectivity
Scramble
shQPRT 1
E
Intra HCV titer
Vector
HA-QPRT
F
Super HCV titer
Vector
HA-QPRT
G
Scr
shQPRT1
HCV
Clo
NS3
Core
QPRT
β-Actin
H
Scramble
shQPRT
GFP-HCV Cell %
Clo
400μM
800μM
HCV

Figure S4

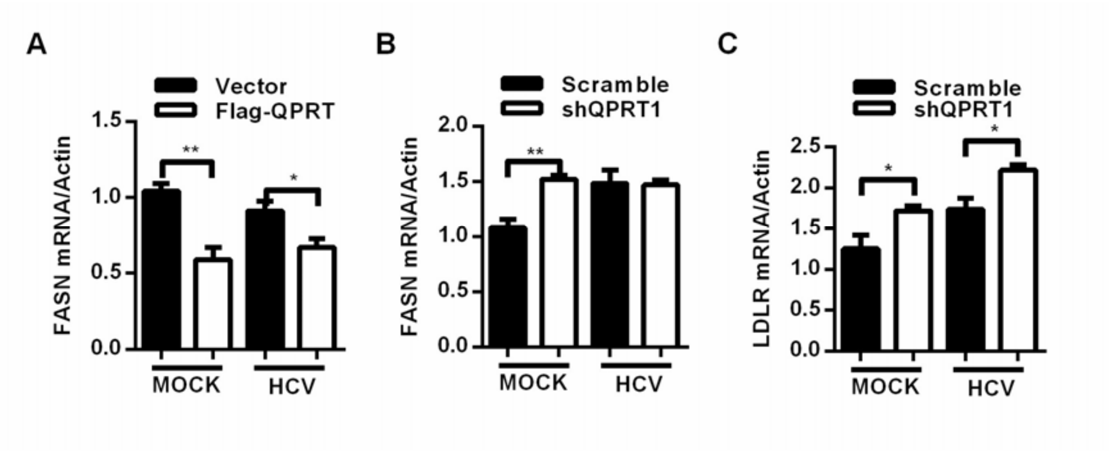

A
Vector
Flag-QPRT
FASN mRNA/Actin
1.5
1.0
0.5
0.0
**
*
MOCK
HCV
B
Scramble
shQPRT1
FASN mRNA/Actin
2.0
1.5
1.0
0.5
0.0
**
MOCK
HCV
C
Scramble
shQPRT1
LDLR mRNA/Actin
2.5
2.0
1.5
1.0
0.5
0.0
*
*
MOCK
HCV

Figure S5

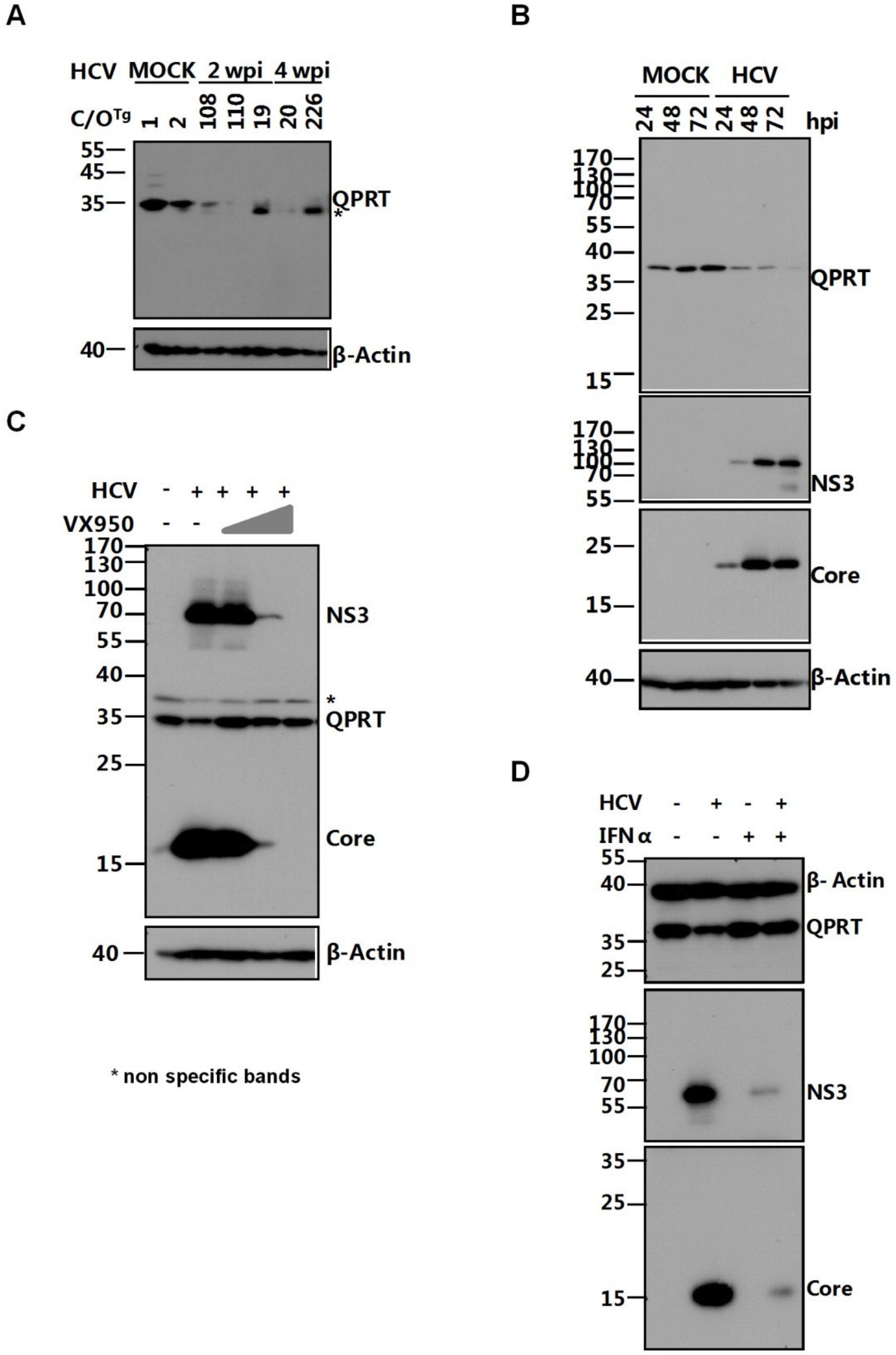
A
HCV MOCK 2 wpi 4 wpi
C/O^Tg 1 2 108 110 19 20 226
55
45
35
QPRT
*
40
β-Actin
B
MOCK HCV
24 48 72 24 48 72 hpi
170
130
100
70
55
40
35
25
15
QPRT
NS3
Core
β-Actin
C
HCV - + + + +
VX950 - -
170
130
100
70
55
40
35
25
15
NS3
*
QPRT
Core
β-Actin
* non specific bands
D
HCV - + - +
IFN α - - + +
β- Actin
QPRT
NS3
Core

Figure S6

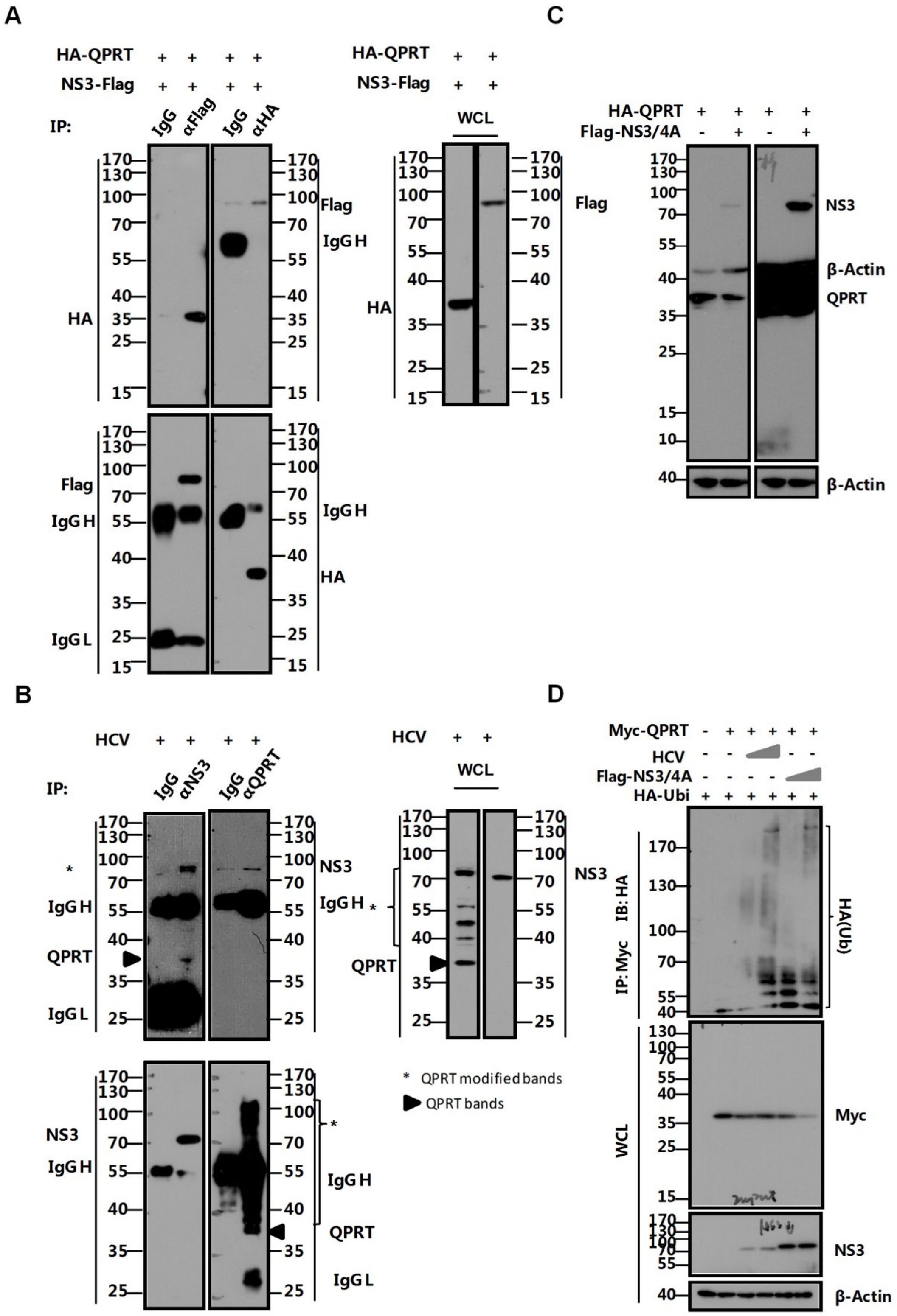
A
HA-QPRT
NS3-Flag
IP:
IgG
αFlag
IgG
αHA
170
130
100
70
55
40
35
25
15
HA
Flag
IgG H
IgG L
HA-QPRT
NS3-Flag
WCL
C
HA-QPRT
Flag-NS3/4A
NS3
β-Actin
QPRT
10
B
HCV
IP:
IgG
αNS3
IgG
αQPRT
*
NS3
IgG H
QPRT
IgG L
HCV
WCL
* QPRT modified bands
QPRT bands
D
Myc-QPRT
HCV
Flag-NS3/4A
HA-Ubi
IP: Myc IB: HA
HA(Ub)
WCL
Myc
NS3
β-Actin

Figure S7

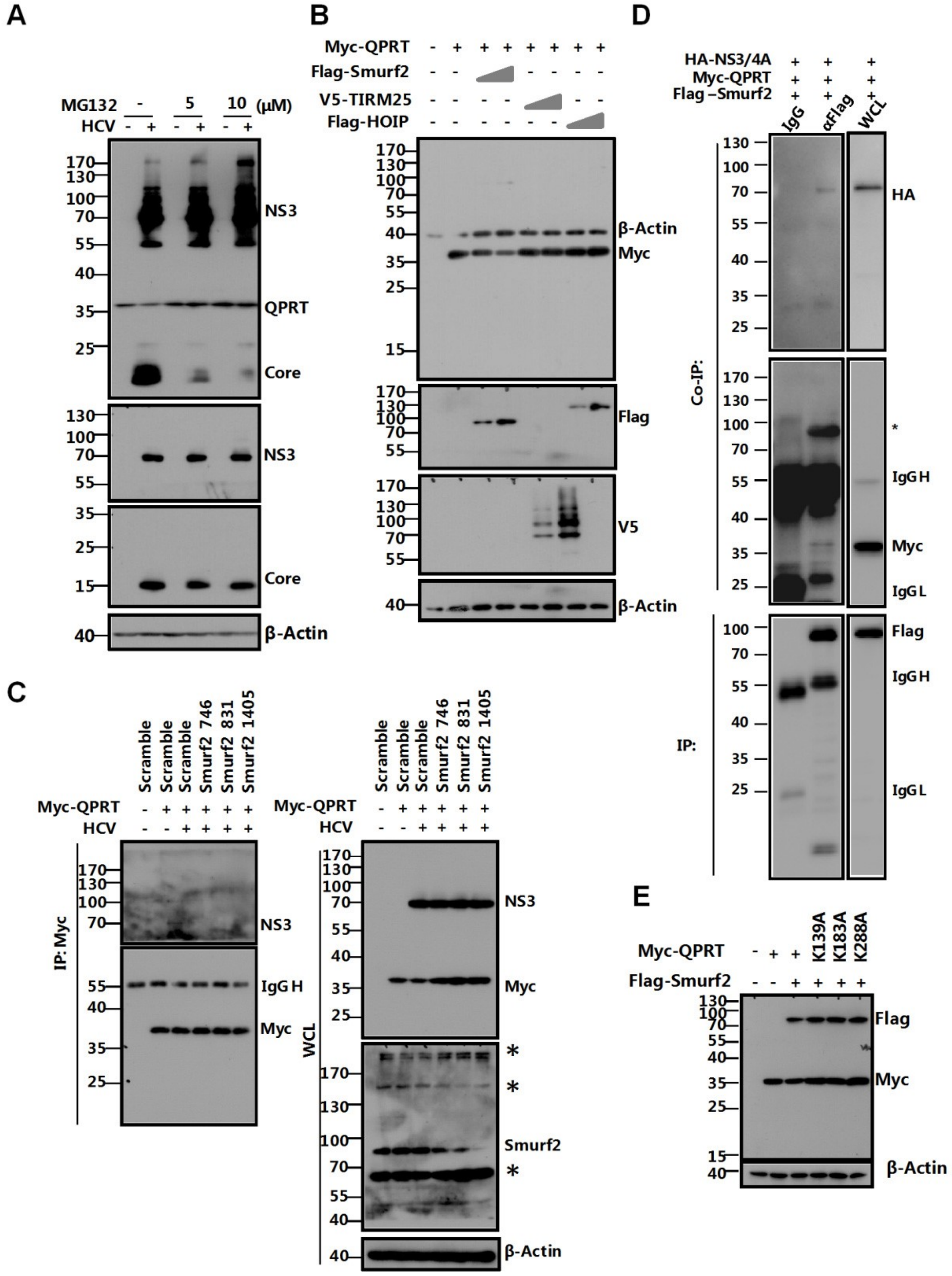

A
MG132 - 5 10 (μM)
HCV - + - + - +
NS3
QPRT
Core
NS3
Core
β-Actin
B
Myc-QPRT
Flag-Smurf2
V5-TIRM25
Flag-HOIP
β-Actin
Myc
Flag
V5
β-Actin
C
Scramble
Scramble
Scramble
Smurf2 746
Smurf2 831
Smurf2 1405
Myc-QPRT
HCV
IP: Myc
NS3
IgG H
Myc
WCL
NS3
Myc
Smurf2
β-Actin
D
HA-NS3/4A
Myc-QPRT
Flag –Smurf2
IgG
αFlag
WCL
HA
Co-IP:
IgG H
Myc
IgG L
Flag
IgG H
IP:
IgG L
E
Myc-QPRT
Flag-Smurf2
K139A
K183A
K288A
Flag
Myc
β-Actin

Figure S8

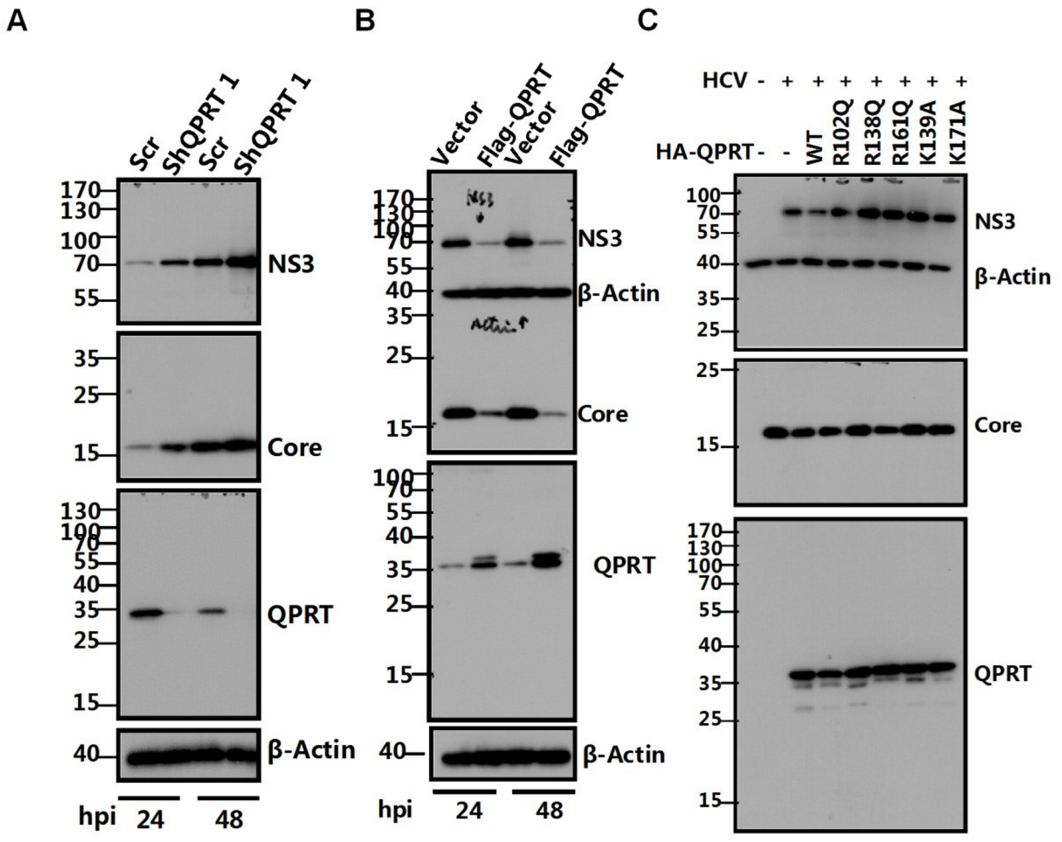
A
Scr
ShQPRT 1
Scr
ShQPRT 1
NS3
Core
QPRT
β-Actin
hpi 24 48
B
Vector
Flag-QPRT
Vector
Flag-QPRT
NS3
β-Actin
Core
QPRT
β-Actin
hpi 24 48
C
HCV - + + + + + + +
HA-QPRT - - WT R102Q R138Q R161Q K139A K171A
NS3
β-Actin
Core
QPRT

Figure S9

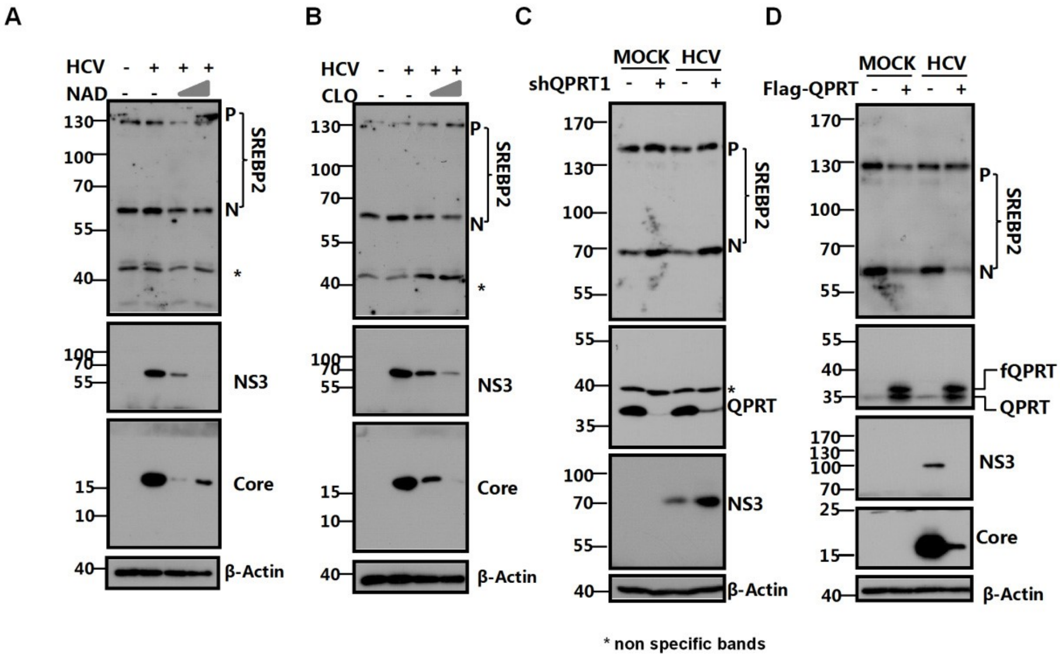
A
HCV - + + +
NAD - - 
130
100
70
55
40
P
N
SREBP2
*
100
70
55
NS3
15
10
Core
40
β-Actin
B
HCV - + + +
CLO - -
130
100
70
55
40
P
N
SREBP2
*
100
70
55
NS3
15
10
Core
40
β-Actin
C
MOCK HCV
shQPRT1 - + - +
170
130
100
70
55
P
N
SREBP2
55
40
35
*
QPRT
100
70
55
NS3
40
β-Actin
* non specific bands
D
MOCK HCV
Flag-QPRT - + - +
170
130
100
70
55
P
N
SREBP2
55
40
35
fQPRT
QPRT
170
130
100
70
NS3
25
15
Core
40
β-Actin

Figure S10

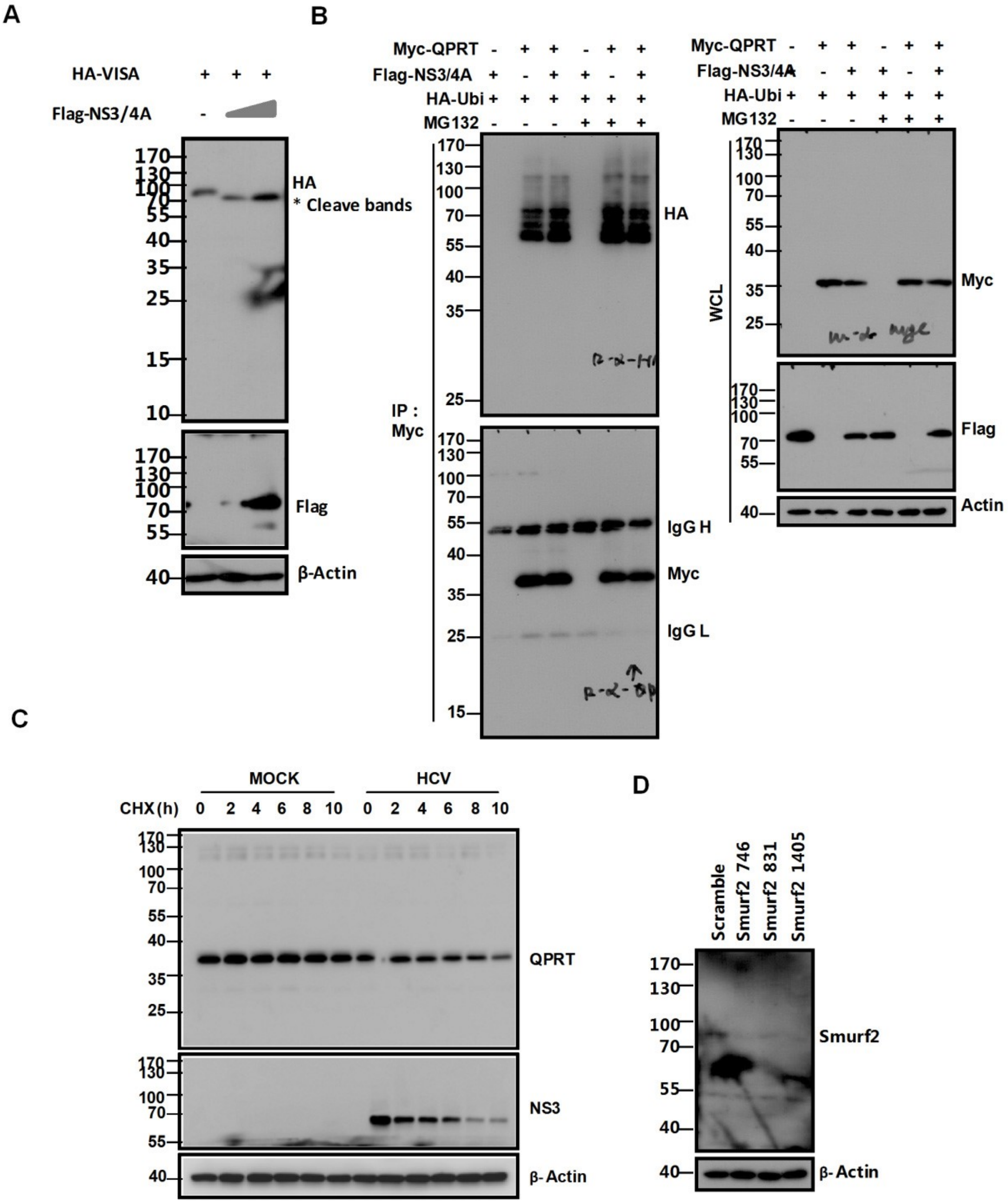
A
HA-VISA + + +
Flag-NS3/4A -
HA
* Cleave bands
Flag
β-Actin
B
Myc-QPRT - + + - + +
Flag-NS3/4A + - + + - +
HA-Ubi + + + + + +
MG132 - - - + + +
HA
IP : Myc
IgG H
Myc
IgG L
WCL
Myc
Flag
Actin
C
MOCK
HCV
CHX (h) 0 2 4 6 8 10 0 2 4 6 8 10
QPRT
NS3
β- Actin
D
Scramble
Smurf2 746
Smurf2 831
Smurf2 1405
Smurf2
β- Actin

Figure S11

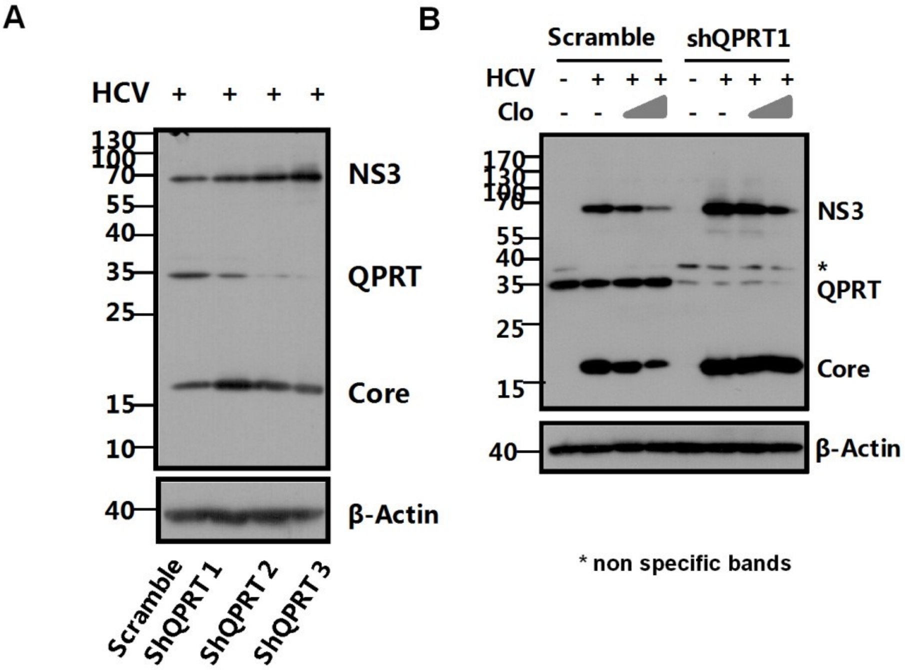
A
HCV + + + +
130
100
70
55
40
35
25
15
10
NS3
QPRT
Core
40
β-Actin
Scramble
ShQPRT1
ShQPRT2
ShQPRT3
B
Scramble
shQPRT1
HCV - + + + - + + +
Clo - - - -
170
130
100
70
55
40
35
25
15
NS3
*
QPRT
Core
40
β-Actin
* non specific bands

**Table S1. Patients characteristics**

| | Disease progress | | |
|---|---|---|---|
| | HV | CHC | Cirrhosis |
| Number of patients | 7 | 11 | 13 |
| Gender (Male/Female) | 1/6 | 6/5 | 9/4 |
| Age | 48.43 ± 1.478 | 47.64 ± 3.550$^{NS}$ | 65.80 ± 3.309 $^{***}$ |
| HCV-RNA >100 IU/ml | ND | 9 | 6 |
| HCV ( S/CO ) >1 | ND | 10 | 13 |
| AST (U/L) | 17.81 ± 1.314 | 53.22 ± 8.543$^{**}$ | 59.52 ± 8.419 $^{**}$ |
| ALT (U/L) | 17.04 ± 3.413 | 60.95 ± 14.67$^{*}$ | 44.49 ± 7.446$^{*}$ |
| Disease grade (G1/G2/G3/G4) | (0/0/0/0) | (3/4/4/0) | (0/0/0/14) |

HV, health volunteer; CHC, chronic hepatitis C; S/CO, signal-to-cutoff ratio in reactive HCV antibody test; ALT, alanine transaminase; AST, aspartate aminotransferase; ND, not detectable.
Data were expressed as mean ± standard deviation. Student *t* test, *$P$<0.05; **$P$<0.01; ***$P$<0.001; NS, not significant.

Table S2. Primer sequences used in RT-PCR

| Species | Gene | Forward primer (5`--3`) | Reverse primer (5`--3`) |
|---|---|---|---|
| Virus | JFH-1 | CTGGGTCCTTTCTTGGATAAAC | TCAGGCAGTACCACAAGG |
| Human | QPRT | GTCCTGCTGGACAACTTCAAGC | TGGGTCAGCATCCCCATGGAG |
| | FASN | AGTTCACGGACATGGAGCACAACA | ATGGTACTTGGCCTTGGGTGTGTA |
| | LDLR | GAATCTACTGGTCTGACCTGTCC | GGTCCAGTAGATGTTGCTGTGG |
| | Smurf2 | TACAGATCCTCGGCTGTCTG | CGATACCACTTGCTGTTGCT |
